# Supplementary material for: Current Updates on the Role of Microbiome in Endometriosis: A Narrative Review
Source: Microorganisms. 2023 Jan 31;11(2):360. doi: 10.3390/microorganisms11020360 (PMC9962481; doi:10.3390/microorganisms11020360)
Supplement: Supplementary file 1 [file microorganisms-11-00360-s001.zip › microorganisms-2142240-supplementary.pdf]

Supplementary Table 1: Study characteristics reporting microbiome changes in endometriosis patients.

References	Year	Study location	Mean age (years old)	Study period	Sample size	Diagnosis	Microbiome analysis
Akiyama et al., 2019	2019	Japan	Endometriosis: 33.9 ± 5.7<br>Control: 32.5 ± 6.0	N/A	Endometriosis: 30<br>Control: 39	Diagnosed via laparoscopic and pathology test (classified by rARSM score: Stage III and IV)	16S rRNA analysis (V5-V6 Regions) on Ion Torrent Platform
Ata et al., 2019 [1]	2019	Turkey	#Endometriosis: 28.5 (range: 26 - 31.3)<br>Control: 27.5 (range: 25.8 - 30)	2016 - 2017	Endometriosis: 14<br>Control: 14	Classified based on the r-ASRM scores	16S rRNA analysis (V3 and V4 region)
Chen et al., 2020 [2]	2020	China	36.07 ± 5.57 (range: 18-45)	April 2018 - February 2019	68 patients:<br>Cervical Canal: 67<br>Posterior Formix: 65<br>Uterine Cavity (Endometrium): 2	Confirmed via laparoscopic and pathology test	16S rRNA analysis (V3-V4 region)
Hernandes et al., 2020 [3]	2020	Brazil	18 - 50	N/A	Endometriosis: 10<br>Control: 11	Confirmed with laparoscopic surgery and histopathology analysis	16S rRNA analysis (V3-V4 region)
Wei et al., 2020 [4]	2020	China	31.47 (range: 23 - 44)	N.A.	Endometriosis: 36<br>Control: 14	Confirmed with laparoscopic surgery and categorized based on r-ASRM	16S rRNA analysis (V4-V5 region)
Perrotta et al., 2020 [5]	2020	Brazil	Endometriosis: 34.9 ± 6.8<br>Control: 35.25 ± 6.9	N.A.	Endometriosis: 35<br>Control: 24	Confirmed by TVUS or MRI or previous surgery	16S rRNA analysis (V4 region)
Lee et al., 2021 [6]	2021	Korea	Endometriosis: 36.20 ± 1.30<br>Control: 39.40 ± 1.10	N/A	Endometriosis: 45<br>Control: 45	Confirmed by histological evaluation and classified according to r-ASRM	16S rRNA analysis (V3-V4 region)
Le et al., 2021 [7]	2021	United States	Endometriosis: 32.5 ± 1.1<br>Control: 32.6 ± 2.0	N/A	Endometriosis: 20<br>Control: 9	Confirmed by laparoscopy/laparotomy and classified according to r-ASRM	16S rRNA analysis (V4 region)

Svensson et al., 2021 [8]	2021	Sweden	Endometriosis: 37.8 (range: 32.8-43.3)<br>Control: 37.0 (range: 32.0-44.0)	September 2016 - N.A.	Endometriosis: 66<br>Control: 198	Confirmed by laparoscopy/laparotomy and classified according to ICD-10 classification of endometriosis	16S rRNA analysis (V1-V3 region) on Illumina HiSeq platform
Wessels et al., 2021 [9]	2021	Canada	Endometriosis: 33.8 ± 5.8<br>Control: 35.1 ± 3.3	2011-2017	Endometriosis: 12<br>Control: 9	Confirmed by laparoscopy and pathology test, according to r-ASRM score	16S rRNA analysis (V3 region) on Illumina MiSeq platform
Huang et al., 2021 [10]	2021	China	Endometriosis: 38.3 ± 7.88<br>Control: 34.0 ± 10.8	June 2019 - October 2019	Endometriosis: 21<br>Control: 20	Confirmed by laparoscopy and pathology test	16S rRNA analysis (V4 region) on Ion Torrent platform
Shan et al., 2021 [11]	2021	China	Endometriosis/Control: 32 ± 2, 32 ± 3	March 2019 - September 2019	Endometriosis: 12<br>Control: 12	Confirmed by histology, according to r-ASRM score	16S rRNA analysis (V3-V4 region) on Illumina MiSeq platform
Chao et al., 2021 [12]	2021	China	Endometriosis: 39.89 ± 6.24<br>Control: 38.23 ± 7.80	July 2017 - December 2018	Endometriosis: 37<br>Control: 66	Confirmed by exploratory laparoscopy or surgical pathology	16S rRNA analysis (V4 region) on Illumina platform
Chang et al., 2022 [13]	2022	Taiwan	Endometriosis: 35.4 ± 6.7<br>Control: N.A.	N.A.	Endometriosis: 23<br>Control: 10	Based on r-ASRM score	16S rRNA analysis (V3, V4, V5 and V9 region) on Illumina platform
Oishi et al., 2022 [14]	2022	Japan	Endometriosis: 37.9 ± 6.4<br>Control: 35.2 ± 8.6	July 2019 - April 2020	Endometriosis: 18<br>Control: 18	Based on r-ASRM score	16S rRNA analysis (V1-V2 region) on Illumina MiSeq platform
Yuan et al., 2022 [15]	2022	China	Endometriosis: 35.28 ± 7.24<br>Control: 33.32 ± 8.04	January 2019 - December 2019	Endometriosis: 36<br>Control: 25	Confirmed via laparoscopy and pathology test (diagnosed as I-IV stages)	16S rRNA analysis (V4 region) on Illumina HiSeq platform
Lu et al., 2022 [16]	2022	China	Endometriosis: 36.75 ± 7.11<br>Control: 35 ± 6.61	30 September 2020 - 31st March 2021	Endometriosis: 16<br>Control: 18	Confirmed by imaging and pathology after surgery, according to r-ASRM score	16S rRNA analysis (V4 region) on Illumina HiSeq platform

References

1. Ata, B.; Yildiz, S.; Turkgeldi, E.; Brocal, V.P.; Dinleyici, E.C.; Moya, A.; Urman, B. The endobiota study: comparison of vaginal, cervical and gut microbiota between women with stage 3/4 endometriosis and healthy controls. *Scientific reports* **2019**, *9*, 1-9.
2. Chen, S.; Gu, Z.; Zhang, W.; Jia, S.; Wu, Y.; Zheng, P.; Dai, Y.; Leng, J. Microbiome of the lower genital tract in Chinese women with endometriosis by 16s-rRNA sequencing technique: a pilot study. *Ann Transl Med* **2020**, *8*, 1440, doi:10.21037/atm-20-1309.
3. Hernandes, C.; Silveira, P.; Rodrigues Sereia, A.F.; Christoff, A.P.; Mendes, H.; Valter de Oliveira, L.F.; Podgaec, S. Microbiome Profile of Deep Endometriosis Patients: Comparison of Vaginal Fluid, Endometrium and Lesion. *Diagnostics (Basel)* **2020**, *10*, doi:10.3390/diagnostics10030163.
4. Wei, W.; Zhang, X.; Tang, H.; Zeng, L.; Wu, R. Microbiota composition and distribution along the female reproductive tract of women with endometriosis. *Ann Clin Microbiol Antimicrob* **2020**, *19*, 15, doi:10.1186/s12941-020-00356-0.
5. Perrotta, A.R.; Borrelli, G.M.; Martins, C.O.; Kallas, E.G.; Sanabani, S.S.; Griffith, L.G.; Alm, E.J.; Abrao, M.S. The vaginal microbiome as a tool to predict rASRM stage of disease in endometriosis: a pilot study. *Reproductive Sciences* **2020**, *27*, 1064-1073.
6. Lee, S.R.; Lee, J.C.; Kim, S.H.; Oh, Y.S.; Chae, H.D.; Seo, H.; Kang, C.S.; Shin, T.S. Altered Composition of Microbiota in Women with Ovarian Endometrioma: Microbiome Analyses of Extracellular Vesicles in the Peritoneal Fluid. *Int J Mol Sci* **2021**, *22*, doi:10.3390/ijms22094608.
7. Le, N.; Cregger, M.; Fazleabas, A.; Braundmeier-Fleming, A. Effects of endometriosis on immunity and mucosal microbial community dynamics in female olive baboons. *Sci Rep* **2022**, *12*, 1590, doi:10.1038/s41598-022-05499-y.
8. Svensson, A.; Brunkwall, L.; Roth, B.; Orho-Melander, M.; Ohlsson, B. Associations between endometriosis and gut microbiota. *Reproductive Sciences* **2021**, *28*, 2367-2377.
9. Wessels, J.M.; Domínguez, M.A.; Leyland, N.A.; Agarwal, S.K.; Foster, W.G. Endometrial microbiota is more diverse in people with endometriosis than symptomatic controls. *Scientific reports* **2021**, *11*, 1-12.
10. Huang, L.; Liu, B.; Liu, Z.; Feng, W.; Liu, M.; Wang, Y.; Peng, D.; Fu, X.; Zhu, H.; Cui, Z., et al. Gut Microbiota Exceeds Cervical Microbiota for Early Diagnosis of Endometriosis. *Front Cell Infect Microbiol* **2021**, *11*, 788836, doi:10.3389/fcimb.2021.788836.
11. Shan, J.; Ni, Z.; Cheng, W.; Zhou, L.; Zhai, D.; Sun, S.; Yu, C. Gut microbiota imbalance and its correlations with hormone and inflammatory factors in patients with stage 3/4 endometriosis. *Arch Gynecol Obstet* **2021**, *304*, 1363-1373, doi:10.1007/s00404-021-06057-z.
12. Chao, X.; Liu, Y.; Fan, Q.; Shi, H.; Wang, S.; Lang, J. The role of the vaginal microbiome in distinguishing female chronic pelvic pain caused by endometriosis/adenomyosis. *Ann Transl Med* **2021**, *9*, 771, doi:10.21037/atm-20-4586.
13. Chang, C.Y.; Chiang, A.J.; Lai, M.T.; Yan, M.J.; Tseng, C.C.; Lo, L.C.; Wan, L.; Li, C.J.; Tsui, K.H.; Chen, C.M., et al. A more diverse cervical microbiome associates with better clinical outcomes in patients with endometriosis: A pilot study. *Biomedicines* **2022**, *10*, doi:10.3390/biomedicines10010174.
14. Oishi, S.; Mekaru, K.; Tanaka, S.E.; Arai, W.; Ashikawa, K.; Sakuraba, Y.; Nishioka, M.; Nakamura, R.; Miyagi, M.; Akamine, K. Microbiome analysis in women with endometriosis: Does a microbiome exist in peritoneal fluid and ovarian cystic fluid? *Reproductive medicine and biology* **2022**, *21*, e12441.
15. Yuan, W.; Wu, Y.; Chai, X.; Wu, X. The colonized microbiota composition in the peritoneal fluid in women with endometriosis. *Arch Gynecol Obstet* **2022**, *305*, 1573-1580, doi:10.1007/s00404-021-06338-7.

16. Lu, F.; Wei, J.; Zhong, Y.; Feng, Y.; Ma, B.; Xiong, Y.; Wei, K.; Tan, B.; Chen, T. Antibiotic Therapy and Vaginal Microbiota Transplantation Reduce Endometriosis Disease Progression in Female Mice via NF-kappaB Signaling Pathway. *Front Med (Lausanne)* **2022**, *9*, 831115, doi:10.3389/fmed.2022.831115.
